# Is Chickpea a Potential Substitute for Soybean? Phenolic Bioactives and Potential Health Benefits

**DOI:** 10.3390/ijms20112644

**Published:** 2019-05-29

**Authors:** Adriano Costa de Camargo, Bruno Trevenzoli Favero, Maressa Caldeira Morzelle, Marcelo Franchin, Emilio Alvarez-Parrilla, Laura A. de la Rosa, Marina Vilar Geraldi, Mário Roberto Maróstica Júnior, Fereidoon Shahidi, Andrés R. Schwember

**Affiliations:** 1 Departamento de Ciencias Vegetales, Facultad de Agronomía e Ingeniería Forestal, Pontificia Universidad Católica de Chile, Casilla 306-22, Santiago, Chile; adrianoesalq@gmail.com; 2 University of Copenhagen, Department of Plant and Environmental Sciences, 2630 Taastrup, Denmark; btf@plen.ku.dk; 3 Department of Food and Nutrition, Faculty of Nutrition, Federal University of Mato Grosso, Fernando Correa Avenue, P.O. box 2367, Cuiabá, MT 78060-900, Brazil; maressamorzelle@hotmail.com; 4 Department of Physiological Sciences, Piracicaba Dental School, University of Campinas, Piracicaba, SP 13414-903, Brazil; marcelo.franchin@yahoo.com.br; 5 Department of Chemical Biological Sciences, Universidad Autónoma de Ciudad Juárez, Anillo Envolvente del Pronaf y Estocolmo, s/n, Cd, Juárez, Chihuahua 32310, México; ealvarez@uacj.mx (E.A.-P.); ldelaros@uacj.mx (L.A.d.l.R.); 6 Department of Food and Nutrition, University of Campinas—UNICAMP, Campinas, SP 13083-862, Brazil; marinavilar35@gmail.com (M.V.G.); mmarosti@unicamp.br (M.R.M.J.); 7 Department of Biochemistry, Memorial University of Newfoundland, St. John’s, NL A1B 3X9, Canada; fshahidi@mun.ca

**Keywords:** legume seeds, genetics, phenolic antioxidants, inflammation, cardiovascular disease, cancer, diabetes, obesity

## Abstract

Legume seeds are rich sources of protein, fiber, and minerals. In addition, their phenolic compounds as secondary metabolites render health benefits beyond basic nutrition. Lowering apolipoprotein B secretion from HepG2 cells and decreasing the level of low-density lipoprotein (LDL)-cholesterol oxidation are mechanisms related to the prevention of cardiovascular diseases (CVD). Likewise, low-level chronic inflammation and related disorders of the immune system are clinical predictors of cardiovascular pathology. Furthermore, DNA-damage signaling and repair are crucial pathways to the etiology of human cancers. Along CVD and cancer, the prevalence of obesity and diabetes is constantly increasing. Screening the ability of polyphenols in inactivating digestive enzymes is a good option in pre-clinical studies. In addition, in vivo studies support the role of polyphenols in the prevention and/or management of diabetes and obesity. Soybean, a well-recognized source of phenolic isoflavones, exerts health benefits by decreasing oxidative stress and inflammation related to the above-mentioned chronic ailments. Similar to soybeans, chickpeas are good sources of nutrients and phenolic compounds, especially isoflavones. This review summarizes the potential of chickpea as a substitute for soybean in terms of health beneficial outcomes. Therefore, this contribution may guide the industry in manufacturing functional foods and/or ingredients by using an undervalued feedstock.

## 1. Introduction

Legumes are a staple food with considerable importance for human nutrition due to their high content of carbohydrates and proteins, especially when diets are plant-based or have restrictions to animal-based products [1]. Historical evidence suggests soybean as a domesticated species that originated in Northeast China between 6000 and 9000 years ago [2,3]. The cultivated soybean (*Glycine max*) is considered to have evolved from the wild species *Glycine soja* [4] and from the transitional evolutionary species *Glycine gracilis*, which is considered a landrace [5]. Xu et al. [6] reported that cultivated soybean with different cpDNA haploytypes originated independently in different zones from different wild gene pools and/or hybrid swarms between wild and cultivated genotypes, evidencing major gene flow from *G. soja* to *G. gracilis* and the latter to *G. max*, with moderate gene contribution from *G. soja* to *G. max* [5,7,8]. The oldest records of soybean cultivation appear in bronze inscriptions and in early writings that date not much earlier than 1100 BC. With the expansion of the Shang dynasty, trade of soybean migrated to South China, Korea, Japan, and South East Asia, where it progressively became a dietary staple [9]. Soybean was introduced to Europe in about 1691, although it became a known food plant only in the 18th century. The introduction of soybean from Europe to the USA occurred around 1804, when its utilization rapidly spread out to the rest of the western world, especially in the 20th century [10]. According to Food and Agriculture Organization (FAO), world production of soybeans is currently around 352.6 million tons, associated to 123.6 million hectares grown globally, with average yields of 3.1 t/ha in 2017, and is presently a major crop in the United States, Brazil, Argentina, India, and China, and the most important legume crop cultivated in the world [11]. In Chile, national soybean production is only for seed exports and not for domestic consumption, and being a secondary producer of this crop worldwide, the cultivated area was 1458 ha of herbicide-tolerant transgenic soybeans in 2017 [12].

Chickpea (*Cicer arietinum*) is the only cultivated species in the genus *Cicer*, and it has never been found in the wild but in the closely related taxa [13]. Recently, *Cicer reticulatum* has been postulated to be the wild progenitor of modern chickpea [13,14]. The area of present-day southeastern Turkey and adjoining Syria is the most probable center of origin of chickpea, which was domesticated with wheat, barley, peas, and lentil as a member of West Asian Neolithic crops during the origin of agriculture around 10,000 years ago in the Fertile Crescent [15], with the oldest available archaeological evidence of chickpea from 7500 BC [16,17]. It is considered to have spread throughout the Mediterranean area about 6000 years ago and to have reached India about 4000 years ago [10], and then to the rest of the world. In chickpea, a particular and drastic narrowing of genetic diversity during domestication has occurred due to a series of bottlenecks unique to this crop [17]. Consequently, chickpea displays a lack of adaptive diversity for an assortment of biotic and abiotic stresses [18]. Unlike cultivated chickpea, wild *Cicer* spp. possess useful variation for several of those traits [19,20,21]. Globally, it is currently cultivated in over 14.5 million ha with an annual production of 14.8 million tons and productivity of around 1 t/ha in 2017 [11], which is very much lower than the estimated potential of 6 t/ha under optimum growing conditions [14]. The top chickpea producing countries in the world are India, Australia, Pakistan, Turkey, and Mexico [11]. In Chile, chickpea is a marginal crop, yielding on average 0.86 t/ha and a surface grown of 409 ha, which is mainly cultivated in the rainfed area of Central Chile, according to the data of the 2015/2016 season [22].

In plants, phenolic compounds are responsible for a plethora of functions, including structural components, UV protection, and antioxidant, signaling, and defense molecules [23]. Lignins and lignans are the phenolic polymers that compose the plant secondary cell wall, giving the physical structure and being tightly related to the growth of different plant parts, mostly stem, roots, and seeds [24]. These polymers are responsible for providing a protective barrier against herbivores, fungi, bacteria, and virus, as well as forming an intricate network of biochemical compounds that play scavenger roles of reactive oxygen species (ROS), antimicrobials, and wound signaling [25,26,27]. Moreover, hydrolyzable tannins are known anti-herbivory molecules, which act by precipitating proteins of the digestive tract of insects, impairing enzyme functions while reducing the digestibility of the ingested proteins [28]. The phenolic acids are important signaling molecules in plant-microbe interactions, for example, salicylic acid, and can improve nutrient uptake and protect against infections [29]. Flavonoids are the main source of plants non-enzymatic antioxidant apparatus; they provide a UV screen effect and play a central role in scavenging ROS derived from photorespiration in high light conditions as well as in low/high-temperature stresses or other oxidative stresses [30]. In addition, anthocyanins display a visual effect of colors ranging from yellow/orange to purple/bluish, which function as an important mechanism of attraction of pollinators and seed dispersers [31,32,33].

Food macronutrients play a crucial role in human nutrition and health. The proximate composition of chickpea and soybean is summarized in Table 1. The same amount of lipids, on a weight basis, provide more than two-fold the energy generated by the intake of proteins and carbohydrates. In this sense, due to its lower lipid content (up to twenty-one-fold less) compared to that of soybean, chickpea stands out as a good option in weight management. Furthermore, the content of insoluble fiber in chickpea is comparable or even higher than that of soybean. Due to their high contents of insoluble fiber, consumption of chickpea and soybean may promote regular bowel movement, thus preventing constipation [34]. Individuals that consume higher contents of fiber may show a lower risk of developing diabetes, cardiovascular diseases, and colorectal cancer [35,36,37]. However, the concept of antioxidant dietary fiber [38] has been gaining attention as several studies support the significant contribution of insoluble-bound phenolics in fruits, legumes, and vegetables and their processing by-products [39,40,41,42,43,44]. Therefore, the transport of dietary antioxidants through the gastrointestinal system was highlighted as an essential function of insoluble fibers [45]. In addition, protein concentrates are potential sources of healthy isoflavones [46]. Although some individuals may be allergenic to chickpea proteins, this feedstock is not consumed as a raw material. Gupta et al. [47] have summarized some industrial and home processed chickpeas and their potential to prevent adverse health effects caused by the presence of allergic components.

According to Valdez and Bolling [55], inappropriate activation and expression of nuclear factor-κB (NF-κB) p65 protein, which leads to inflammation, has been found to be positively correlated with the severity of inflammation and neutrophil inflammation in biopsies from inflammatory bowel disease patients. In addition, a recent study [56] has demonstrated that phenolic extracts containing phenolic acids and flavonoids were able to decrease the activation of NF-κB. The literature provides sufficient evidence on the link between oxidative stress and NF-κB activation [57,58]. Accordingly, it has been proposed that screening the antioxidant potential of plant food phenolics could be considered a first step in the studies aiming to prospect novel food bioactive, functional ingredients, and/or nutraceuticals [56]. Besides scavenging ROS, an “ideal antioxidant” should not only be a good reducing agent but must exhibit chelating capacity, and some isoflavones fit these characteristics. Therefore, as for the antioxidant potential, this review focuses on isoflavonoids from chickpea and soybean, the latter being the reference feedstock. When appropriate, the remaining phenolic compounds are cited to support a discussion on the role of structure/activity. The health benefits are discussed in terms of the prevention of cardiovascular diseases (CVD), certain types of cancer, diabetes, and obesity, among others, as inflammation and oxidative stress are common to all of them. In short, this contribution may guide the industry in manufacturing functional foods and/or ingredients by using chickpea which, thus far, is an undervalued feedstock.

## 2. Genetics

Although the benefits of phenolic compounds (e.g., phenolic acids and flavonoids), in food, feed, cosmetics, and medicine are well known, the genetic basis regulating their content in edible seeds is still not fully understood [59,60,61,62,63]. Isoflavones are the main compounds contributing to the phenolic profile of soybeans (2n = 40 chromosomes) [64]. Unraveling the genetics underlying their accumulation in seeds has been difficult because of several factors. Soybean has many quantitative trait loci (QTL) with small individual effects involved in an additive fashion that affects the phenotype and, by consequence, the accumulation of secondary metabolites [65,66,67]. Cai et al. [60] found that *qIF5-1* can explain 49–52% of the isoflavone accumulation in Huachun 2 × Wayao F_7:8–10_ recombinant inbred lines, still far from being an easy trait to be determined and in line with other authors [59] who list several small-effect impact of QTLs on isoflavone seed content. However, in this intricate network, Li et al. [68] found four multidrug and toxic compound extrusion (MATE) transporter-encoding genes in wild soybean that increased antioxidant content and are unrelated to the phenylpropanoid pathway, which indicates phenolic intercellular transportation processes with a potential to be determinant. Besides, soybean possesses a complex genome that has passed by several whole genome duplication events [69,70]. Another concern stems from the fact that isoflavone content is strongly modulated by environmental conditions during seed development, thus affecting their synthesis and accumulation [69,71]. The variation of a warmer to a normal year in a two-year trial planted in eastern Maryland reduced isoflavones by c.a. 50% due to the higher temperature, while a cooler central location on the same state was not affected [72]. Individual identification and quantification of 12 different isoflavones of soybean seeds (including the most cited glycitein, daidzein, and genistein) have been technically difficult, cumbersome, and expensive, with liquid chromatography coupled to spectrophotometric detection being the most commonly used technique [60,73]. In order to properly determine the underlying genetic nuances of isoflavone accumulation, it is pivotal to have the most common compounds consistently determined and preferably in a high throughput setup. In addition, epistatic interactions, that is, the particular combination of alleles that results in a change in the phenotype, meaning that the same allele can produce different phenotypes in a different genetic background, are responsible for a great proportion of the observed phenotypic variance [74,75]. Furthermore, adding complexity to QTL analysis for validating isoflavone accumulation involves multiple interactions between those QTLs with different environmental variables that affect this trait [76,77]. The literature shows contrasting values of the heritability, that is, the proportion of observed phenotypic variability among individuals that is due to genetic differences among them, for isoflavones content of soybean seeds. These heritability values range between lower than 40% [66,67,78] and higher than 80% [79,80]. Thus, in the latter case, indicating that most of the variation among genotypes is explained by genetic effects allows to fine map major QTLs associated with both individual and total isoflavone content in soybean seeds, that could potentially be introgressed into elite cultivars through marker-assisted selection (MAS) [81].

The first study of DNA markers related to isoflavone content of soybean seeds used the recombinant inbred line (RIL) population ‘Essex’ × ‘Forrest’, and the markers associated to this trait was reported by Njiti et al. [82]. Using the same RIL population, subsequent works identified new QTLs associated with genistein, glycitein, and daidzein on six different soybean chromosomes [65,78,83]. Another RIL population ‘AC756’ × ‘RCAT Angora’ was employed for the identification of QTLs associated with genistein, glycitein, and daidzein [66]. In parallel, the same major QTL (on chromosome 7) was detected in another population based on the parental lines ‘Zhongdou 27’ × ‘Jiunong 20’ [67]. Other two major QTLs associated to total isoflavone content, genistein, glycitein, and daidzein were reported on chromosomes 5 (*QDZGT1*) and 8 (*QDZGT2*), by utilizing the ‘Hwangkeum’ (*Glycine max*) × ‘ITI82932’ (*Glycine soja*) population [84]. Other groups developed additional RILs populations (‘Essex’ × ‘PI 437654’, ‘Magellan’ × ‘PI 437654’) with the objective to identify major isoflavone-associated QTLs, and coincided with the previous reported QTLs, although several isoflavones-associated QTLs were detected in these studies [59,75,76]. This research group identified five QTLs that contributed to the concentration of isoflavones, possessing single or multiple additive effects on isoflavone component traits, and a major locus on chromosome 5 (Gm05) was validated that alone accounted for up to 10% of the phenotypic variance for glycitein, and 35–37% for daidzein, genistein, and the sum of all three soybean isoflavones. This Gm05 QTL was consistently associated with isoflavones concentration across different crosses, locations, and years [76]. More recently, a high-density soybean genetic map was constructed using RILs (Luheidou2 × Nanhuizao, F_5:8_), and 41 QTLs were identified that contributed to the isoflavone content, standing out one novel major QTL on chromosome 20, *qIF20-2*, which contributed to a majority of isoflavone components across various environments and explained a high amount of phenotypic variance (up to 35.3%) [85]. Using specific-locus amplified fragment sequencing in the F_5:7_ of the same RIL cross, Pei et al. [86] found four new QTLs (*qG8*, *qMD19*, *qMG18*, and *qTIF19*) associated to genistin, malonyldaidzin, malonylgenistin, and total isoflavones, respectively. Meanwhile, another study identified 44 isoflavone-associated QLTs using soybean landraces and restriction site-associated DNA tag sequencing (RAD-seq), including 55 candidate genes on 16 chromosomes, explaining 72.2% of the total phenotypic variation, reflecting the complex genetic nature of the synthesis and accumulation of isoflavones on soybean seeds [87]. Subsequently, 15 stable QTLs associated to individual and total isoflavone content were identified using a high-density soybean genetic map across multiple environments of China, by utilizing a population of 196 F_7:8–10_ RILs (Huachun 2 × Wayao). In this study, one major QTL on chromosome 5, *qIF5-1*, contributed significantly to the total isoflavone content and explained between 43.3 and 52.5% of the total isoflavone content phenotypic variance [60]. Taking all together, the research results of the last 20 years have generated knowledge for a better understanding of the genetics of isoflavone accumulation in soybean seeds, especially with the fact that there are genetic regions on chromosome 5 that appear to play an important role in the regulation of this trait. In addition, there is scope available for improvement of isoflavone content through MAS [88], which could be a powerful tool to increase isoflavone content by the introgression of valuable QTLs/genes/alleles (i.e., identified on chromosome 5) into elite cultivars of soybean that would certainly augment their seed quality. Another promising way of finding candidate genes that could further be validated by QTL is the use of genome-wide association studies (GWAS). Recently, an MYB transcription factor was found to regulate isoflavone contents in soybean hairy roots, the *GmMYB29* activated the promoters *IFS2* and *CHS8*, while its overexpression or silencing by RNAi had positive and negative effects on isoflavone content [89]. Nonetheless, further studies to verify and confirm biosynthetic pathways using RNA-sequencing and co-expression network analysis can lead to the identification of specialized varieties, for example, producing coumestrol [90].

Contrary to soybean, there is very little information in the literature on the role of genetics with respect to the polyphenolic composition of chickpea (2n = 16 chromosomes) [91], and to the best of our knowledge, there is no study associated with the genetic regulation of isoflavones on chickpea seeds. One important field of research in the last decades has been the alteration of isoflavone contents and composition upon germination [92]. However, the available literature demonstrates that variations in the isoflavone contents and profile are dependent on the process [93,94]. Changes of the isoflavones content upon germination has very likely a genetic regulation explanation, although no QTLs/genes associated with this phenomenon have been reported so far. Most of the genetic studies of chickpea have focused on the identification of genetic regions using different methodologies associated to improving agronomic traits, such as increasing seed yield and the yield of components [95,96,97,98,99,100,101,102,103]. In addition, a genetic approach has been used to develop drought/heat tolerance [70,104,105,106,107,108], as well as disease resistance, for example, Ascochyta blight [109,110]. Solid and broad genetic studies on chickpeas are emerging and paving the way for breeding approaches targeting phenolic compounds in general [111,112,113]. From a nutritional and functional food perspective, genetic studies have been carried out on chickpeas to identify genetic regions associated to seed color [114,115,116], seed protein content [117], and seed-zinc/iron concentrations [118]. Based on the existing literature, it seems clear that increasing the levels of isoflavones on chickpea seeds by germination could have valuable implications, to gain insight into the genetic basis regulating this process, which would be of scientific interest to identify QTLs/genes/alleles of relevance that could be transferred into elite chickpea cultivars through MAS and/or traditional breeding. In addition, genetic comparative studies between soybeans and chickpeas are of importance to understand whether the existing knowledge of the genetics underlying the regulation of isoflavone synthesis in soybeans might be transferable to chickpea.

## 3. Main Phenolic Bioactives and Their Quantities

In addition to being rich sources of carbohydrates, protein, fibers, minerals, and vitamins, chickpea, soybean, and other legumes also contain bioactive substances [119,120,121,122,123]. Phenolic compounds are present in both soluble and insoluble-bound forms. Therefore, one should bear in mind that optimizing the extraction of phenolics in the soluble form is desirable (Table 2).

Xu and Chang [121] carried out a comparative study on the yield of phenolics of legumes as affected by the extraction solvent. Their results support the use of 50% acetone from chickpea and yellow soybean while black soybean showed a higher total phenolic content (TPC) when acidic 70% acetone (0.5% acetic acid) was employed. The contrast found by these authors might be related to differences in specific phenolics present. Supporting this assumption, it is worth to highlight the study carried out by Yoshiara et al. [120]. These researchers used a centroid design and made a valuable contribution by demonstrating that glycosidic isoflavones were better extracted with the polar ternary mixture (water, acetone, and acetonitrile, 2:1:1, *v*/*v*/*v*) while malonyl-glycosidic isoflavones showed higher extraction yields with mixtures of water, acetone, and ethanol (2:1:1, *v*/*v*/*v*). Finally, water and acetone (1:1, *v*/*v*) rendered a higher extraction of isoflavones as aglycones. Examples for the total phenolic contents (TPC) of soybean and chickpea are summarized in Table 2, while the isoflavone profile of chickpea and soybean is summarized in Table 3.

Chickpea and soybean have significant amounts of flavonoids, especially isoflavones. According to the literature, the total isoflavone content (TIC) varied from 153 to 340 mg/100 g of chickpea and from 165 to 336 mg/100 g of soybean [51,126]. In chickpea, the major isoflavones found were biochanin A and formononetin while smaller amounts of genistein and daidzein might also occur [94]. Considering the contribution of each isoflavone to TIC, the isoflavone profile of chickpea and soybean is summarized in Table 3. Formononetin and biochanin A, which are present in chickpeas, have not been reported in soybeans. In addition, the presence of phenolic acids and other flavonoids in chickpeas were reported by Thavarajah and Thavarajah [133]. However, isoflavonoids have been reported as the main bioactive phenolics of chickpea [93]. As for the absolute concentration, biochanin A (180 μg/g) and its derivatives, biochanin glucoside (80 μg/g), and biochanin A glucoside malonylated (60 μg/g) made the highest contribution to the isoflavone profile of chickpeas, while formononetin (100 μg/g) rendered a lower contribution [93]. The same study [93] also reported the concentration of daidzein (120 μg/g) and genistein (60 μg/g). It is important to highlight that these concentrations were in good agreement with those found in soybeans, which were in the range of 18.5–242.7 μg/g for daidzein and 13.0–158 μg/g for genistein, which suggests that chickpeas may be considered a good substitute for soybeans as a source of isoflavone aglycones [132]. Finally, it is noteworthy that current identification and quantification of isoflavones in these feedstocks were mainly carried out using hyphenated techniques, such as liquid chromatography coupled to tandem mass spectrometry (LC–MS^n^), thus increasing the reliability of the results. 

## 4. Potential Health Benefits

### 4.1. Antioxidant Potential

Dietary phytochemicals, specifically phenolic compounds, are known by their potency in scavenging ROS. Likewise, the antioxidant activity of phenolic compounds may be explained by their reducing power and/or metal-chelating activity towards ferric and ferrous ions, respectively. The Fenton reaction, also known as the Haber-Weiss cycle, is an important biological model. Peroxyl and hydroxyl radicals may be pointed among the most important ROS as both participate in the Fenton reaction. The role of ferric and ferrous ions in the Fenton reaction has also been addressed [23]. Peroxyl radicals show a longer half-life compared to that of hydroxyl radicals. Consequently, while hydroxyl radicals may damage intracellular components, the deleterious effects of peroxyl radicals may be extended to biological fluids [137,138].

The antioxidant properties of soybean are mainly associated with the presence and/or profile of isoflavones. Furthermore, a highly positive correlation existed between the concentration of genistein in phenolic extracts of soybean and their ability in scavenging 2,2′-azino-bis(3-ethylbenzothiazoline-6-sulphonic acid) diammonium salt (ABTS) radical cation [130]. The presence of genistein as aglycone has been found in only one sample (out of seven) in Egyptian cultivars of chickpeas as evaluated by RP-HPLC-DAD-ESI-QTOF-MS (RP: reversed-phase; DAD: diode array detection; ESI: electrospray ionization; QTOF: quadrupole time of flight; MS: mass spectrometry), while its conjugated counterpart was detected in all samples [139]. The experience of our group with various feedstocks has demonstrated that the chelating ability of polyphenols is not simple to be confirmed, which is the opposite of the reducing power as, in general, the latter has been correlated with TPC [137,138].

As for the structure/activity, phytochemicals with one or more methoxy substitutions are regarded as potent chelating agents [140]. In contrast, compounds lacking catechol or galloyl moiety do not show any complex formation [141]. The properties of genistein and biochanin A as metal chelators have been confirmed by elemental analysis, Fourier-transform infrared spectroscopy, thermogravimetric analysis, and electrospray ionization mass spectrometry [142]. However, daidzein does not chelate Cu(II) or Fe(III), thus supporting a structure/activity relationship. These authors [142] also suggested that isoflavones bind metals at the 4-keto and the 5-OH sites. Additionally, it has been hypothesized that the ratio of ferric to ferrous ion is important for rapid initiation of lipid peroxidation through the Fenton reaction and the ratios of 1:1 to 7:1 (Fe^3+^/Fe^2+^) are optimum [143]. Therefore, reducing the concentration of ferric ions in the system could be beneficial. However, due to their cyclic nature, ferrous ions are oxidized to the ferric form, and the latter is again reduced to the ferrous form. In this scenario, it has been suggested that an “ideal antioxidant” should not only be a good reducing agent but must exhibit chelating capacity [23], hence genistein and biochanin A could be classified as ideal antioxidants.

Trolox, a water-soluble analog of vitamin E, is used as a standard to express the antioxidant activity as evaluated by several methods (e.g., 2,2-diphenyl-1-(2,4,6-trinitrophenyl)hydrazyl (DPPH), ABTS, ferric reducing antioxidant power (FRAP), oxygen radical absorbance capacity (ORAC)). Therefore, by examining the antioxidant activity in the available literature (Table 4), especially from the same research team and feedstock, it is easy to understand that the antioxidant activity should not be faced as a single number but rather as an index and/or trend. Yoshiara et al. [130] evaluated the antiradical activity of soybean seeds. The antioxidant potential towards DPPH radical and ABTS radical cation was 289 ± 1.0 and 252 ± 6.0 µmol Trolox equivalent/g, respectively. In general, these differences have been explained by their operative mechanisms. In fact, the ability of phenolic compounds in scavenging free radicals stems from single electron transfer (SET) or hydrogen atom transfer (HAT). Furthermore, the same authors [130] also showed that, regardless of the method, germination increased the antioxidant properties of the test material.

A deeper evaluation of the data reported by Yoshiara et al. [130] shows that the greatest increase (up to 138%) was found against ABTS radical cation compared to the improvement towards DPPH radical (up to 12%). Likewise, while a significant difference between the antioxidant activity (ABTS assay) of free and insoluble-bound phenolics of soybean was found by Ademiluyi and Oboh [129], these authors did not find any difference with respect to the reducing power. Therefore, these studies [129,130] demonstrate that specific phenolics (e.g., conjugated versus isoflavones as aglycones) or fractions (free versus insoluble-bound phenolics) respond differently to each method. Additionally, besides the degree of glycosylation, the number and position of hydroxyl groups in polyphenols may also influence their antioxidant potential [23,126].

Beyond the starting material, their fractions (e.g., cotyledons, epicotyls, radicles, and hypocotyls) may show different antioxidant properties [130]. According to Sreerama et al. [119], the antioxidant activity towards hydrogen peroxide and DPPH radical was in the decreasing order of seed coat > embryonic axe > cotyledons. Therefore, evaluating the whole material, that is, along with non-edible portions, may not provide the best picture of the potential antioxidant activity under physiological conditions. Like plant foods, the human body relies on antioxidant systems to eliminate free radicals and the excessive increase in their levels can damage proteins, lipids, and nucleic acids. In addition, oxidative stress and lipid peroxidation are major causes of chronic ailments, such as neurodegenerative, inflammatory, and cardiovascular diseases. Likewise, the role of ROS in allergies, immune system dysfunctions, type 2 diabetes, obesity, certain types of cancer, and aging have been reported [146,147].

In humans, endogenous antioxidant enzymes, such as catalase (CAT), superoxide dismutase (SOD), glutathione peroxidase (GPx), glutathione reductase (GR), and peroxiredoxins (PRXs), exert antioxidant function during oxidative stress [148,149]. Phenolic compounds may also modulate the activity of antioxidant enzymes. Genistein has been found to improve the activity of antioxidant enzymes, as well as decreasing the levels of ROS and lipoperoxide in the brain and liver of C57BL/6J streptozotocin (STZ) diabetic mice, thus reverting the overproduction of ROS and restoring glutathione content and the reduced glutathione (GSH) and oxidized glutathione (GSSG) ratio [150]. Although these authors [150] did not address the bioaccessibility and further bioavailability of genistein, the improved oxidative status of brain and liver found by them suggests that the test compound was bioavailable. It is well accepted that isoflavones as aglycones are usually more bioavailable than their conjugated counterparts. The bioaccessibility and bioavailability of several phenolic compounds have recently been revised and published elsewhere [151]. Furthermore, genistein also increased the activity of hepatic superoxide dismutase, catalase, and glutathione peroxidase of STZ-induced diabetic rats [152]. Genistein and daidzein decreased malondialdehyde (MDA) production against anoxia-glucopenia and reperfusion damage in rat urinary bladder (A-G/R) [153], thus supporting their antioxidant activity in vivo.

Biochanin A, which is found in chickpeas [154], has been gaining attention due to its therapeutic value. Biochanin A reduces oxidative stress by decreasing MDA levels and by increasing the levels of CAT, SOD, and total antioxidant status in diabetic rats [155]. Exposure to arsenic through the consumption of contaminated drinking water and foods may cause significant negative health effects, including cancer and non-cancerous diseases. Jalaludeen et al. [140] tested the therapeutic efficacy of biochanin A against arsenic-induced renal and cardiac damage in rats. According to these authors, Biochanin A alleviated oxidative stress in the kidney of arsenic-treated rats. In addition, biochanin A exhibited a protective effect against oxidative stress in a rat model of Parkinson’s disease, inhibiting the nicotinamide adenine dinucleotide phosphate oxidase (NADPH oxidase) activation and MDA production and increasing SOD and GPx activities in the rat brain [156].

### 4.2. Anti-Inflammatory Effects

During oxidative stress, interleukins and inflammatory mediators are produced. Isoflavones from natural products have shown anti-inflammatory properties in vitro and in vivo [157]. Verdrengh et al. [158] showed that subcutaneous treatment with genistein was able to suppress the delayed-type hypersensitivity reaction to oxazolone and the granulocyte-mediated response. Likewise, Zhang et al. [159] demonstrated that genistein treatment inhibited the proliferation of rheumatoid synoviocytes by reducing the matrix metallopeptidase 9 (MMP-9) expression [159]. Genistein may also prevent periodontal disease. Bhattarai et al. [160] tested this hypothesis in a mice model of periodontitis. According to these authors, genistein was able to protect against alveolar bone loss and periodontal tissue degradation. Neuroinflammation, a phenomenon that is primarily mediated by microglial cells, may culminate in Parkinson and Alzheimer disease, multiple sclerosis, amyotrophic lateral sclerosis, among other chronic ailments. The potential of genistein in treating and/or preventing neuroinflammation has already been discussed [161]. The modulatory effects induced by flavonoids are mediated by their impact, mainly, on mitogen-activated protein kinase (MAPKs) and nuclear factor-kappa B (NF-κB) [161]. Lending support to the statements of Spagnuolo et al. [161], Ji et al. [162] demonstrated that tumor necrosis factor alpha (TNF-α) and interleukin 6 (IL-6) release in lipopolysaccharide (LPS)-stimulated macrophages was inhibited upon genistein treatment via NF-κB, specifically by stimulating adenosine monophosphate-activated protein kinase (AMPK). It has been reported that in vitro LPS-stimulated microglia cells (BV2) liberate less nitric oxide, prostaglandin E2, IL-1β, and TNF-α after genistein treatment [163]. In this scenario, the authors also attributed the inhibitory effects of genistein on the NF-κB. Furthermore, genistein reduced the binding of the LPS to the toll-like receptor 4 (TLR-4) receptor on microglial cells [163].

Biochanin A, a derivative of genistein, is also able to reduce neuronal inflammatory damage of the articular cartilage [164,165]. Wu et al. [166] showed that biochanin A attenuated LPS-induced pro-inflammatory responses and inhibited the activation of the MAPK pathway in BV2 microglial cells and decreased the expression of TNF-α and interleukin 1 beta (IL-1β). Therefore, the mechanisms of action of biochanin A are attributed to its ability in reducing phosphorylation of MAPK, c-Jun N-terminal kinases (JNK), extracellular signal-regulated kinase (ERK), and p38 [166]. However, biochanin A acts simultaneously on other targets. Ming et al. [167] reported that biochanin A suppressed the rolling and adhesion molecules expression, specifically the vascular cell adhesion molecule 1 (VCAM-1), intercellular adhesion molecule 1 (ICAM-1), and E-selectin molecules on human umbilical vein endothelial cells. The effects of biochanin A are related to the activation of Peroxisome proliferator-activated receptor (PPAR)-γ. Furthermore, its ability to reduce NF-κB activation has also been reported [167]. E-Selectin reduction is a marker for endothelial function improvement, controlled metabolic condition, and arteriosclerosis and inflammation reduction in diabetic patients [168,169].

Daidzein, another isoflavone aglycone, also shows anti-inflammatory potential [170]. According to Ahmad et al. [171], daidzein reduced the evolution of rheumatoid arthritis in Wistar albino rats. These authors showed that the levels of TNF-α and rheumatoid arthritis scores were improved when compared to the control group. Furthermore, the same study suggested protective effects towards CVD as it reduced the levels of low-density lipoproteins and triacylglycerols. Beneficial effects of daidzein in the lung were also observed in mice. Feng et al. [172] used an LPS-induced acute lung injury model. The test material reduced important inflammatory parameters, that is, the number of infiltrated cells and levels of inflammatory cytokines in bronchoalveolar lavage fluids. The inhibition of TLR4-MyD88-NF-κB signaling pathway is possibly involved in the protective action of daidzein. NF-κB and signal transducer and activator of transcription 1 (STAT-1) routes are important targets related to nitric oxide production, an important inflammatory mediator [173]. Besides reducing the NF-κB route, other authors have also shown that daidzein and other flavonoids (e.g., kaempferol and quercetin) are able to simultaneously affect the STAT-1 route [173]. Penga et al. [174] attempted to improve the pharmacokinetic properties of daidzein. These authors synthesized new sulfonic acid ester derivatives with much better pharmacologic effects than the original molecule. Furthermore, the derivatives inhibited phosphorylation of JNK and showed significantly enhanced anti-inflammatory activities by two to four orders of magnitudes over that of the parent daidzein in TNF-α-stimulated Caco-2 cells.

Formononetin, a daidzein derivative, has in vivo antinociceptive effects in mice models and reduced the neutrophil migration to the peritoneal cavity and hind paw edema [175]. Formononetin was also evaluated using an LPS-stimulated lung injury model on mice and showed promising protective effects. Ma et al. [176] showed that formononetin treatment reduced the bronchoalveolar lavage fluid cell numbers, increased PPAR-γ gene expression, and improved the superoxidase dismutase activity while simultaneously inhibited the myeloperoxidase activity. Wu et al. [177] evaluated the effects of formononetin on dextran sulfate sodium- (DSS-) induced acute colitis model in mice. Dose-dependent attenuation of colitis was noted, and, according to these authors, the effects of formononetin may stem from the inhibition of NLRP3 inflammasome signaling pathway.

### 4.3. Polyphenols in the Prevention of CVD

A recent report stated that CVD is the most common non-communicable ailment, claiming one-third of total global death [178]. In general, low-density lipoprotein-cholesterol (LDL-c) levels are predictors of death from cardiovascular diseases (CVD) [179]. Development of atheromatous Aplaques stems from the uptake of oxidized LDL-c, via scavenger receptors, thus leading to cholesterol accumulation and foam cell formation [23]. Therefore, it is important to highlight that oxidized LDL-c is the actual entity involved in the early event of atherosclerosis. Tarantino et al. [180] carried out a study with obese patients and showed that altered copper bioavailability is a predictor of early atherosclerosis, the main CVD risk in obese patients with hepatic steatosis. Accordingly, cupric ion-induced human LDL-c peroxidation in vitro has been employed to anticipate the potential benefits of polyphenols in reducing the risk of CVD, although some authors have used 2,2′-azobis (2-methylpropionamidine) dihydrochloride (AAPH) to induce the oxidation process [181].

The generation of conjugated dienes and trienes, as well as malondialdehyde-thiobarbituric acid (MDA-TBA) or TBA reactive substances (TBARS), have been used to monitor the level of LDL-c oxidation [181,182]. Genistein (200 μmol/L) has shown inhibitory activity by protecting LDL-c from peroxyl radical (azo-initiated)-induced oxidation in vitro [183]. Kerry and Abbey [183] supported their results in the lower time required (3 h) for MDA formation compared to that of the control (7 h) as well as evidenced by a decrease in relative electrophoretic mobility (REM) of LDL-c.

Xu et al. [184] carried out a comparative evaluation on the ability of common food legumes, including soybeans and chickpea, towards copper-induced human LDL-c oxidation in vitro. Total phenolics and total flavonoids were correlated (r = 0.79 to 0.94, *p* < 0.001) with the antioxidant activity in this model system, and the authors suggested that phenolic compounds played a possible role in the overall antioxidant activity of legumes. In addition, high correlations existed between ORAC assay (scavenging of peroxyl radical) and the protection of LDL-c against oxidation (r = 0.81 to 0.83, *p* < 0.001).

The potential of dietary chickpeas in reversing dyslipidemia in rats induced by a chronic high-fat diet was studied by Yang et al. [185]. Chickpea supplementation (10% *w*/*w*) induced a favorable lipid profile by decreasing the levels of triacylglycerols, LDL-c, and LDL-c:HDL-c ratio. Pittaway et al. [186] suggested that dietary supplementation with chickpeas results in lowering total serum and LDL-c levels in hypercholesterolemic women and men as compared to a wheat-supplemented diet. Improvements in serum lipid profile (e.g., lower serum total cholesterol and LDL-c) were confirmed in another study from the same research team [187]. These authors [185,186,187] did not evaluate the phenolic profile of their starting material. However, the role of phenolics in cardiovascular diseases is well substantiated [188]. Supporting the role of legumes in the prevention of CVD, Sedaghat et al. [189] carried out a clinical trial and demonstrated that soybean consumption decreased the level of total serum cholesterol and LDL-c in patients with type 2 diabetes. Other clinical trials [190,191] showed similar results with respect to the role of soybean, their products and related phytochemicals in reducing total cholesterol and LDL-c levels, thereby improving the cardiovascular risk profile.

According to the literature [192], cellular cholesterol synthesis was inhibited by genistein (41%) and daidzein (18%), and the effect was associated with increases in 3-hydroxy-3-methylglutaryl-CoA reductase mRNA. After univariate analysis, Pittaway et al. [187] concluded that dietary fiber exerted the greatest single effect by reducing serum total cholesterol, but increasing pieces of evidence show that dietary fibers act as carriers of phenolic antioxidants [45]. Lipopolysaccharides (LPS) are present in the cell wall of Gram-negative bacteria. Intestinal barrier dysfunction due to high intake of saturated fatty acids facilitates LPS migration into the bloodstream, thus enabling systemic inflammation, which is common to cardiovascular disease. Furthermore, phenolic acids and flavonoids from legume crops show antimicrobial activity and protect barrier integrity [193,194]. Therefore, phenolic compounds may be helpful in the prevention of CVD by inhibiting the growth of Gram-negative bacteria, protecting barrier integrity [193,194], and possible LPS release into the bloodstream, in addition to their well-documented anti-inflammatory action [157]. 

### 4.4. Polyphenols as Adjuvants in Cancer Prevention and Treatment

An unhealthy diet is among the risk factors responsible for cancer, a leading cause of death worldwide. Colorectal, female breast, liver, lung, and stomach cancer are among the most common causes of cancer death [195]. Lung cancer is most common in men, while breast cancer is most prevalent in women. Different from other chronic ailments (e.g., obesity and diabetes), clinical trials which are reporting the effects of soybean, chickpea, and other legumes in cancer development and/or management are not available. However, a meta-analysis of epidemiologic studies supports the negative correlation between soybean intake and lung [196] as well as breast cancer [197].

Girón-Calle [198] investigated the cell growth-regulating properties of chickpea seed extracts as possible responsible anti-cancer factors. Their results demonstrated that chickpea extracts inhibited the growth of Caco-2 cells exhibiting a cancerous phenotype. The effects of biochanin A on cell growth in the mammary carcinoma cell line MCF-7 has also been reported [199]. The preventive action of phenolic compounds against lung, liver, and female breast cancer is dependent on the bioaccessibility of the compounds as these must reach the plasma and different tissues. In contrast, stomach and colorectal cancer, which are also major causes of cancer death, do not necessarily stem from the bioaccessibility of polyphenols. Insoluble-bound phenolics have been found in soybean and other legume seeds [200]. Insoluble-bound phenolics are not readily bioaccessible. However, it has been hypothesized that upon human colonic microbiota fermentation, insoluble-bound bioactive phenolics can be released in the colon [200], thus potentially preventing colorectal cancer.

DNA-damage signaling/repair are crucial pathways to the etiology of human cancers [201]. In fact, it has been well accepted that DNA strand breakage may culminate in mutagenesis and affect replication and transcription of DNA, which are pointed among the causes of cancer initiation [23]. The presence of phenolic extracts from different legume seeds inhibits peroxyl radical-induced DNA damage [202,203]. Soluble and insoluble-bound fractions of black soybean seed coat and cotyledon contain isoflavones as aglycones, as well as their conjugated forms [204]. Phenolics from back soybean seed coat showed antioxidant activity by preventing AAPH-induced oxidative DNA-damage in HepG2 cells as noted by the inhibition of formation of 8-hydroxy-2′-deoxyguanosine (8-OHdG) as a biomarker for oxidative stress [203].

Mycotoxins, as chemical carcinogens, induce DNA-damage in cell models as well as in vivo [205,206]. The occurrence of aflatoxins, deoxynivalenol, and zearalenone has been reported in soybeans [207,208], while chickpea and chickpea-based products tested positive for aflatoxin and ochratoxin A [209]. However, several studies [210,211,212,213,214,215] have demonstrated that phenolic compounds protected against mycotoxin-induced dysfunctions (e.g., aflatoxins, deoxynivalenol, and zearalenone) in animal and cell models. Therefore, as a source of phenolic compounds, soybeans, chickpea, and their processing by-products may counteract potential health concerns caused by mycotoxins.

The role of transcription factors, such as NF-κB, AP-1, and STAT3 and their gene (e.g., tumor necrosis factor, interleukin-1, interleukin-6, chemokines, cyclooxygenase-2, 5 lipoxygenase, matrix metalloproteases, and vascular endothelial growth factor, adhesion molecules, and others), in inflammation and cancer has been well discussed in the available literature [216]. Acrylamide is probably carcinogenic to humans. Accordingly, chickpea flour has been used as a strategy to decrease the formation of acrylamide in processed foods (e.g., baked goods and fried potato) [217]. Some authors [217,218] have suggested that the beneficial effects of protein hydrolyzates stem from the ability of chickpea and soybean peptides in reacting with acrylamide, thus forming new derivatives. However, another study [219] has supported the ability of flavonoids (e.g., daidzin, genistin, daidzein, and genistein) in reducing acrylamide formation, which was correlated to the number of phenolic hydroxyl groups of flavonoids, their antiradical activity, and reducing power. 

Surgery, chemotherapy, radiation, or their combination are the most common treatments for cancer [195]. Furthermore, the oxidative stress-based hypothesis involving the production of ROS due to the use of anticancer drugs has gained acceptance [220]. Side effects of chemotherapy include cardiotoxicity, hepatotoxicity, nephrotoxicity, neurotoxicity, and gastrointestinal and pulmonary toxicity [221]. In addition, hepatotoxicity and cardiotoxicity may be linked to drug-induced oxidative stress [220,222]. Anthracycline (ANT), which has been used in the treatment of leukemias, breast, and lung cancer, go through redox cycling where ROS are generated [220] due to the presence of ANT-Fe^2+^ complexes. The lower oxidative stress in anthracycline-treated rats, compared to control, was attributed to treatment with phenolic compounds [222].

The antioxidant activity and radioprotection isoflavone against gamma-irradiation were investigated by Dixit et al. [223]. These authors concluded that soy isoflavone scavenged free radicals induced by gamma-irradiation exposure and inhibited radiation-induced cellular damages. They also found increased survival rate of the animals pretreated with soy isoflavone and improved hematological and histological parameters as compared to the irradiated control group. Singh et al. [224] evaluated the effect of X-ray irradiation exposure on human lymphocytes isolated from the peripheral blood in vitro and suggested an age-related decline in DNA repair competence. The potential of genistein in preventing DNA damage, chromosomal aberration, and apoptosis triggered by X-ray irradiation in HL-7702 (L-02) cells was studied by Song et al. [225]. According to them, the molecular mechanism underlying its radioprotective properties might be explained by the inhibition of the endoplasmic reticulum stress marker GRP78, the promotion of the expression of HERP, a multifunctional protein, and the up-regulated expression of HUS1 and hHR23A, which are DNA repair-related genes. Therefore, consumption of soybeans and chickpea as natural sources of antioxidants may play an adjuvant’s role in cancer treatment.

### 4.5. Polyphenols in the Management and/or Treatment of Type 2 Diabetes and Obesity

Overweight (BMI [body mass index] 25–29.9) and obesity (BMI > 30) are defined by the World Health Organization (WHO) as abnormal or excessive fat accumulation that may impair health. Their fundamental cause is an imbalance between energy intake and energy expenditure, and their prevalence is on the rise around the world. From the 7500 million world population, it is estimated that 774 million are obese, and more than 50% of them live in the USA, China, or India [226]. Type II diabetes mellitus, which represents at least 90% of all diagnosed cases of diabetes, is one of the major diseases commonly associated with obesity. Diabetes is the fourth or fifth cause of death in the developed world, and together with obesity, they are projected to rise if measures are not taken to diminish this escalation [227]. Several strategies have been used to control and reduce these two diseases, among them dietary interventions, increased physical activity, medication, and consumption of functional foods and/or nutraceuticals are important. However, employing a combination of several strategies is probably more effective. Soybeans and chickpeas contain several kinds of bioactive compounds that may have positive effects on the control of obesity and type II diabetes. Thus, it is important to describe and discuss the main findings in this research area.

#### 4.5.1. Digestive Enzymes as Biochemical Targets of Bioactive Compounds

Food macronutrients (carbohydrates, lipids, and proteins) must be hydrolyzed, and their hydrolysis products are absorbed through the digestive tract before they can be used as a source of energy and biosynthetic precursors in the tissues of higher animals. Many secondary metabolites present in soybeans, chickpeas, and other pulses are potent inhibitors of the enzymes responsible for the hydrolysis of food macronutrients. Protease inhibitors are especially abundant and may be considered as antinutritional factors because they reduce protein digestibility and hence, diet quality. They can have a negative health impact, especially in monogastric animals and in protein-restricted diets, but their effects can usually be eliminated or, at least, reduced by cooking, since many protease inhibitors are themselves proteins or peptides [228,229]. Other antinutritional factors, such as phytic acid, reduce mineral bioavailability by forming insoluble complexes with metal ions.

Likewise, inhibitors of the enzymes responsible for dietary carbohydrate and lipid hydrolysis used to be considered as antinutritional factors as they could reduce the bioavailability of these high energy nutrients enough to cause growth inhibition [228]. However, in recent times, the capacity of secondary metabolites in inhibiting carbohydrate and lipid hydrolysis has been viewed as a beneficial property. In fact, reducing the absorption of these macronutrients has been pointed out as one of the mechanisms by which some bioactive compounds decrease the risk of developing obesity and type II diabetes. Phenolic compounds are among the secondary metabolites that, in addition to their many biological activities, are being recognized as inhibitors of digestive enzymes. They can inhibit protein, carbohydrate, and lipid hydrolysis to different degrees, depending on several factors, including the structure of the enzyme as well as the phenolic compounds [230,231,232].

Starch (composed of two distinct polysaccharides, amylose and amylopectin) is the most abundant dietary carbohydrate. It is broken-down to monosaccharides (glucose) through the action of two types of enzymes present in the human digestive tract: α-amylases and α-glucosidases. There are two isoforms of human α-amylase: salivary and pancreatic. They both hydrolyze internal α1→4 glycosidic linkages of starch, yielding short-chain oligosaccharides. Pancreatic α-amylase, which is secreted by the pancreas but acts in the small intestine, carries out most of the starch hydrolysis (70 %) and its final products are mainly maltose, maltotriose, and dextrins (fragments of amylopectin containing α1→6 branched units). Human pancreatic α-amylase, a 55 kDa enzyme, contains 496 amino acid residues organized in three structural domains (A, B, and C). Domain A contains the catalytic triad (Asp197, Glu223, and Asp300) inside a (β/α)_8_ barrel [231].

Intestinal α-glucosidases act upon the products of pancreatic α-amylase, producing free glucose that is absorbed through specific transporters in the membrane of enterocytes. They consist of four different individual enzymes organized in two complexes: maltase-glucoamylase (MGAM) and sucrase-isomaltase (SI). Each complex possesses a molecular weight of approximately 260 kDa and contains a C-terminal (Ct) luminal domain and an N-terminal (Nt) domain covalently linked with a transmembrane protein of the intestinal brush border. Each domain has a distinct catalytic activity: MGAM Ct domain is glucoamylase and Nt domain is maltase, SI Ct domain is sucrase and Nt domain is isomaltase [233]. All four enzymes exhibit exo hydrolytic activity towards the non-reducing ends of α1→4 glycosidic linkages, but each catalytic unit possesses unique enzymatic characteristics. For example, glucoamylase (MGAM Ct) has the highest α1→4 hydrolytic activity, while isomaltase (SI Nt) can also hydrolyze α1→6 bonds [234]. α-Glucosidases from different origins, including those from rat and mouse intestine as well as yeast and bacterial α-glucosidases, have been used in studies aiming to screen potential α-glucosidase inhibitors [235]. Therefore, comparison among them may be difficult.

**Figure 1:**
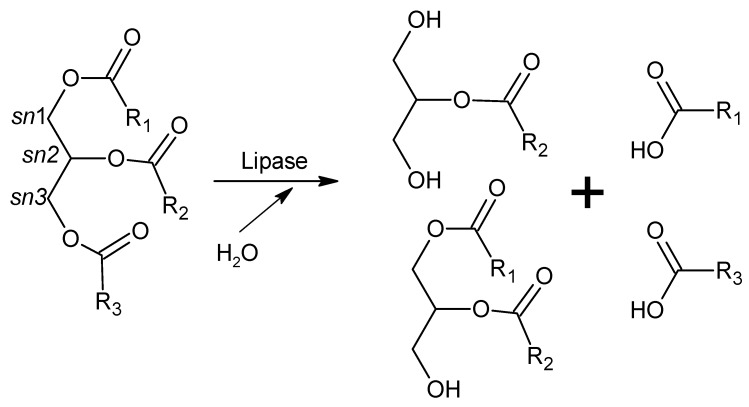
Hydrolysis of triglyceride by lipase activity. R_1_, R_2_, and R_3_ are fatty acid residues.

Fatty acids, which are the major lipid fuels of animal cells, are released from dietary triacylglycerols (found in fats and oils) by the action of intestinal lipases and then diffuse into the enterocytes crossing their apical membrane and are used as energy sources in many tissues. Although two pre-duodenal lipases are present in the human digestive system (lingual and gastric lipases), pancreatic lipase (secreted by the pancreas but acting in the small intestine) is the main enzyme responsible for lipid absorption (70%) [231]. Pancreatic lipase releases fatty acids from *sn*1 and *sn*3 positions of dietary triacylglycerols, yielding monoacylglycerols, diacylglycerols, and free fatty acids (Figure 1). It is a 50 kDa, 449-amino acid protein with two structural domains (Ct and Nt). The catalytic triad (Ser152, Asp176, and His263) is located in the Nt domain, while the Ct domain contains the interaction site with colipase [231]. Colipase is a small protein (10 kDa), also secreted by the pancreas, that acts as a cofactor required for the activity of pancreatic lipase in the presence of bile salts. Colipase anchors pancreatic lipase to the lipid/water interface of the micelles formed between bile salts and long-chain triacylglycerols, allowing it to display its catalytic activity [236].

#### 4.5.2. Phenolics from Soybean and Chickpea as Inhibitors of α-amylase, α-glucosidase, and Lipase

Few studies have explored the inhibition of carbohydrate and lipid hydrolases by chickpea and soybean phenolic compounds. Most have only worked with phenolic-rich extracts, and just a few have thoroughly characterized the extracts and identified the bioactive compound(s). In Table 5 and Table 6, the main findings of these studies, most of which have focused on α-glucosidase (Table 5) and α-amylase (Table 6), are summarized.

The inhibition of both carbohydrate hydrolases (α-amylase and α-glucosidase) by phenolic extracts of chickpea [125,127] and soybean [129,237] has been studied in the last decades. All these studies found a similar inhibitory activity for both enzymes, except McCue et al. [237] who found that soybean extracts were better inhibitors of α-amylase than α-glucosidase. The chickpea extract studied by Sreerama et al. [119,127] contained phenolic acids and flavonoids, such as flavonols, isoflavones, and proanthocyanidins, although the authors did not identify the most bioactive component responsible for enzyme inhibition. The chickpea extract had lower α-glucosidase inhibitory activity and intermediate α-amylase inhibitory activity, compared with other legumes (cowpea [*Vigna unguiculata*] and horse gram [*Macrotyloma uniflorum*]), and it was a better inhibitor of both enzymes than the soybean extracts studied by Ademiluyi and Oboh [129]. The studies of Sánchez-Magaña et al. [125] and McCue et al. [237] showed that fermentation increased the enzyme inhibitory activity of chickpea and soybean phenolic extracts, respectively.

Several studies have analyzed the inhibition of α-glucosidases by phenolic-rich chickpea and soybean extracts. Yao et al. [131] compared the α-glucosidase inhibition (as a percentage) elicited by phenolic-rich ethanolic extracts of chickpea, soybean, and other legumes, such as peas and different kinds of beans. The chickpea extract was found to be a better α-glucosidase inhibitor than the soybean extract, but both showed considerably lower activity than extracts of various bean species, of which Adzuki bean (*Vigna angularis*) had the highest activity. The authors found no correlation between α-glucosidase inhibition and phenolic acid content or antioxidant activity [131]. This lack of correlation was also reported by Tiwari et al. [238], who studied the effect of sprouting on the α-glucosidase inhibitory activity, antioxidant, and other biological activities of chickpea extracts. Interestingly, the authors found mitigation of postprandial glycemic response in rats pretreated with extracts (sprouted and non-sprouted) of one chickpea variety, but this effect was not correlated with the α-glucosidase inhibitory activity [238]. In contrast, Lee et al. [132] did find a correlation between phenolic content of soybean extracts obtained with different solvent polarity (different methanol content, acetone, dichloromethane) and α-glucosidase inhibition. Authors observed the highest α-glucosidase inhibitory activity in the 70% methanol extract (higher than acarbose, positive control), which also presented the highest total phenol content and individual isoflavones (mainly glucosides, malonylglucosides, and acetylglucosides). In another study, Lee et al. [239] evaluated the effect of fermentation on ten varieties of soymilk over the inhibitory activity against α-glucosidase and lipase. Fermentation increased the inhibitory activity toward both enzymes, and the authors observed a correlation between total phenolic compounds and enzyme inhibition. As expected, upon fermentation, glycosylated isoflavones content decreased while aglycone content increased. Other studies have found that food products containing chickpea and soybean also possess α-glucosidase inhibitory activity. Furthermore, treatments like cooking, sprouting, or fermentation generally increases the capacity of the phenolics obtained from these feedstocks, inhibiting the activity of the aforementioned enzyme [132,238,239,240,241]. None of the studies analyzed the inhibitory mechanisms of the extracts or explored the molecular interactions between α-glucosidase and the chickpea or soybean bioactive.

Most studies published to date on the inhibition of α-amylase by chickpea extracts have used water as a solvent and considered the presence of inhibitors as an antinutritional factor. However, the extracts have generally shown low inhibition of α-amylase (as compared with other pulses, such as soybean and other beans), which is further decreased by cooking, suggesting the presence of peptide inhibitors and a low antinutritional potential of chickpea [243,244].

The work of Moussou et al. [245] found a moderate inhibitory activity of a chickpea water extract, which was slightly enhanced by thermal treatment; therefore, although they did not identify the inhibitory compounds in the extract, their heat-stability could indicate the presence of phenolic compounds. The authors also highlighted the health-promoting potential of α-amylase inhibitors, for their ability to avoid a rapid rise in postprandial glucose, which is a risk factor for the development of diabetic complications, in opposition to their antinutritional effects, which would be evident only in case of excessive α-amylase inhibition.

Shi et al. [243] compared the α-amylase inhibitory activity of aqueous extracts of chickpea, soybean, and other pulses (peas, lentils, and beans) and found that only beans (*Phaseolus vulgaris*) and soybean possessed this activity. The authors also found high protease inhibitory activity in the soybean extracts, but this was due to the presence of inhibitory peptides, which are significantly degraded upon cooking [243]. Another study showed that the α-amylase inhibitory activity of soybean aqueous extracts increased during fermentation, depending on the time of fermentation and the species of microorganism [246].

Inhibition of pancreatic lipase by chickpea and soybean has been less studied. In Table 7, the main findings of these studies are summarized. Lee et al. [247] studied the inhibition of aqueous methanolic extracts obtained from defatted chickpea and soybean flours. Although the authors did not identify the bioactive compounds in the extract, aqueous methanol was one of the preferred solvents for the extraction of phenolic compounds. The chickpea and soybean extracts showed similar EC_50_ values, slightly lower than that of mung bean (*Vigna radiata*), but in the range of red bean (*Vigna angularis*) and black-eyed pea (*Vigna unguiculata*). Interestingly, the activity of chickpea was not affected by simulated in vitro digestion. Two studies that analyzed the effect of fermentation on soybean anti-lipase potential found a decrease in glycosylated flavonoids (including isoflavones), an increase in isoflavone and other flavonoid aglycones, and an increase in lipase inhibitory activity in the extracts of fermented soy products [239,248]. This indicated that the anti-lipase activity of aglycones was superior to that of glycosides.

Tan et al. [242] carried out a detailed study on the inhibition of the three digestive enzymes (α-glucosidase, α-amylase, and lipase) by crude, semi-purified, and fractionated phenolic extracts, as well as pure phenolic compounds of black soybean and other foods. They found that the soybean crude and semi-purified extracts were among the most potent inhibitors of the three enzymes with greater activity against α-glucosidase > lipase > α-amylase. In addition, some extract fractions rich in total phenolic compounds, flavonoids, and condensed tannins were better α-glucosidase and α-amylase inhibitors than commercially available inhibitors. Correlations were found between total phenol and total flavonoid content and IC_50_ values against α-glucosidase; and between condensed tannin content and IC_50_ against α-amylase. The authors observed that phytochemical content and antioxidant activity were not the only predictors for enzyme inhibition and suggested that some specific structures might have a stronger inhibitory effect on certain enzymes [242].

#### 4.5.3. Phenolic Compounds and Digestive Enzymes and Their Structure-Activity Relationship (SAR)

As described in the previous section, the published studies regarding the inhibition of digestive enzymes by chickpea and soybean phenolic compounds are scarce, and their inhibition results are only reported as percentage of inhibition, half maximal inhibitory concentration (EC_50_), or enzymatic inhibition index, without giving information on the inhibition kinetics or the mechanism of action. However, considering the abundance of total and individual phenolic compounds found in chickpeas and soybeans, it is possible to propose the mechanism of action of these phenolic compounds as digestive enzyme inhibitors.

Some authors have summarized the possible mechanisms of action of phenolic compounds on the inhibition of digestive enzymes. They have also proposed the main structure-activity relationship (SAR) that regulates these inhibitory processes [231,249,250,251,252,253,254]. Even though, in some cases, semi-purified extracts have been used to evaluate the inhibition patterns of phenolic compounds, in order to propose the inhibitory mechanisms, it is preferable to evaluate the inhibitory activity of pure phenolic compounds since synergistic or antagonistic effects can be observed among them or with other compounds, such as sugars or organic acids, normally present in phenolic extracts, sometimes even at higher concentrations than the phenolic compounds [250,255].

In general terms, it is accepted that the inhibitory activity of phenolic compounds against digestive enzymes depends on two main factors: (i) structural characteristics of the enzyme, including hydrophobicity, isoelectric point, size, and amino acid composition [252]. A higher inhibition of α-amylase (55 kDa) than α-glucosidase (260 kDa) has been reported by some authors, suggesting that the size of the enzyme plays an important role in their inhibition by phenolic compounds [125,256]. Phenolic compounds are more active toward carbohydrate-hydrolyzing enzymes, compared to lipases, probably because lipases are active in a less polar environment [231]. Extraction solvent polarity also plays an important role in enzyme inhibition by phenolic compounds. In this context, Benatti Justino et al. [257] reported that phenolic extracts obtained with low polarity solvents (butanol, ethyl acetate) showed higher inhibitory activities toward the three digestive enzymes compared to extracts obtained with polar solvents (water, ethanol). In another study, Tan et al. [242] observed that while amylase and glucosidase enzymes were highly inhibited by condensed tannins extracted from soybeans, lipase was better inhibited by flavonoids rich extracts. (ii) structural characteristics of phenolic compounds, including molecular weight, number of hydroxyl groups, glycosylation, and hydrogenation [231,252]. It has been extensively reported that phenolic acids show lower inhibitory activity than flavonoids and that the activity of phenolic acids increases with the number of hydroxyl groups present in their structure [231]. In the case of polymeric compounds (condensed tannins), oligomeric structures (<10 monomeric units) show higher inhibitory activity compared to polymeric structures (>10 monomeric units) and monomeric phenolic compounds, probably because oligomeric structures are able to form specific interactions with the active site of the enzyme, while bigger structures form non-specific interactions within all the enzyme surface [258] and monomeric compounds form less or weaker interactions. Interestingly, a synergic effect of monomeric phenolic compounds and condensed tannins with different degree of polymerization from black bean extracts has been reported for the inhibition of α-amylase, α-glucosidase, and lipase [242].

In order to gain information on the inhibitory mechanism of action of phenolic compounds against digestive enzymes, kinetic studies, in combination with other techniques, such as fluorescence spectroscopy, calorimetric studies, and in silico analyses by molecular docking of pure compounds, are necessary. The inhibition of digestive enzymes by phenolic compounds is mainly due to spontaneous (ΔG −) reversible non-covalent specific interactions [231,250], even though in the case of high molecular weight proanthocyanidins (condensed tannins), non-specific covalent and non-covalent interactions may occur [258]. Hydrophobic interactions, hydrogen bonds, van der Waals, and electrostatic interactions are the main forces involved in the phenolic compounds-digestive enzymes interactions [231,250]. Hydrophobic interactions (ΔH +, ΔS +) occur mainly between two aromatic groups (π stacking interactions), one from the phenolic compound, and the second one from an aromatic residue in the enzyme. van der Waals forces (ΔH −, ΔS −) are present between the aromatic ring of the phenolic compound and methyl groups or aliphatic chains in the enzyme. Hydrogen bonds (ΔH −, ΔS −) occur between hydroxyl groups from the phenolic compound and oxo or hydroxyl groups in the enzyme. Finally, electrostatic interactions occur between hydroxyl groups from the phenolic compound and charged amino groups from Lys or Arg residues [231,250]. The number and intensity of these non-covalent interactions are regulated by physicochemical parameters, such as temperature, solvent polarity, pH, and saline concentration.

The main inhibition mechanisms reported for phenolic compounds as inhibitors of digestive enzymes is mixed-type inhibition, in which both competitive (ki) and non-competitive (ki’) components are present [231,250,259,260], indicating that the phenolic compound may bind both near the binding site and/or in another region of the enzyme and that they can bind both the free enzyme and the enzyme-substrate complex. Flavonoids have shown mixed-type inhibition of both α-amylase and lipase, in which the competitive component (inhibitor binds tighter with free enzyme) regulates the process [259,260]. Similar results were observed for caffeic and *p*-coumaric acids with lipase [259].

Structure-activity relationship (SAR) is an analytical (qualitative or quantitative) technique that allows us to determine the association between the structure of bioactive compounds to their biological/chemical activities. For phenolic compounds, it has been used to determine the association between their main structural characteristics (number and position of hydroxyl groups, number of conjugated double bonds, methylation, glycation, galloylation) and their antioxidant, antiproliferative, and enzyme inhibitory activities. SAR analysis combines the kinetic, spectroscopic, and in silico modeling (molecular docking) results in order to better understand the main structural characteristics of phenolic compounds that allow them to inhibit the digestive enzymes. With these studies, it is possible to propose the binding site as well as the type of interaction present between the enzyme and the different phenolic compounds.

SAR analysis has been used mainly to describe the structural characteristics of flavonoid-enzyme interactions, because, as previously described, phenolic acids showed lower inhibitory activity toward digestive enzymes. Table 8 summarizes the main structural characteristics of flavonoids that affect their inhibitory activity of α-amylase, α-glucosidase, and lipase [231,249,250,251,252,253,254,261].

As shown in Table 8, the unsaturation of the C2–C3 bond in combination with the 4-oxo group (both in ring C), which provides rigidity and planarity to the flavonoid structure, increases the inhibitory activity toward the three digestive enzymes. In this context, flavan-3-ol (catechin), which lacks both the double bond at C2–C3 and 4-oxo group, showed neither lipase nor α-amylase inhibitory activity [231,260]. 

Increasing the number of hydroxyl groups in all rings is another relevant characteristic for enzyme inhibition. This inhibitory effect is more evident when ortho diphenols (catechol) are present in the structure. Substitution of hydroxyl groups in any of the rings for methoxy or glycoside moieties drastically reduces the enzymatic activity due to the loss of hydrogen bonds and steric hindrance. Interestingly, when this glycoside moiety is replaced by a phenolic acid (gallic acid), the α-glucosidase activity of the flavonoids is increased [253]. Finally, the substitution of C3 hydroxyl group in ring C of flavonoids by a galloyl moiety increases the enzymatic inhibition of the three digestive enzymes, due to the increase in the number of hydroxyl groups in the molecule.

Few SAR studies have been carried out to evaluate the effect of isoflavones against digestive enzymes [249,251,253,254,262]. Tadera et al. [262] reported lower inhibition of α-glucosidase than α-amylase in the presence of daidzein (2 hydroxyl groups at positions 7 and 4’) and genistein (3 hydroxyl groups at positions 5, 7, and 4’). They also observed that the inhibition of both enzymes was higher with genistein, indicating that hydroxylation of isoflavone ring A increased the inhibitory activity against α-amylase and α-glucosidase. In the case of ring B, no systematic effect of hydroxylation was observed. Methoxylation of hydroxyl groups in all rings decreased the inhibitory activity of isoflavones. The same effect was observed upon glycosylation of hydroxyl groups in ring C [253,254].

In order to confirm the structure-activity relationship and the mechanism of action of phenolic compounds in digestive enzyme inhibition, in silico studies by molecular docking and molecular potential analyses are performed. Docking analysis predicts the possible binding sites between phenolic compounds and digestive enzymes [258,260]. For this, the minimal free energy change (ΔG) and the structure of ligand (phenolic compound)-protein complex is determined, in order to propose the most favorable binding pockets and ligand conformation. Docking results predict the main interactions between amino acid residues with phenolic compounds, as well as the main non-specific covalent interactions present in the complex formation. Docking results confirm that the main interactions between digestive enzymes and monomeric phenolic compounds (phenolic acids and flavonoids) occurred near the active site, through hydrophobic interactions, hydrogen bonds, van der Waals, and electrostatic interactions, in agreement with kinetic and spectroscopic results. Interestingly, docking results obtained between proanthocyanidin oligomers (heptamers) and α-amylase showed multiple interaction sites with the surface of the enzyme through numerous hydrophobic interactions, instead of one single interaction site with the catalytic cavity [258]. Similar results were obtained for the interaction of proanthocyanidin oligomers with lipase. Trimer and tetramer proanthocyanidins interacted in the lipase-colipase binding region through hydrogen bonding, resulting in a higher inhibition compared to a heptamer proanthocyanidin, which interacted only with colipase in a region not critical for the lipase-colipase union, necessary to catalyze the triacylglycerol hydrolysis [258].

From the analysis of the information given in the present review, it is evident that both chickpea and soybean may inhibit digestive enzymes by the action of monomeric and polymeric phenolic compounds present in their matrix. However, further studies are necessary in order to be able to identify the individual inhibitory compounds and propose their mechanism of action against digestive enzymes, as well as to evaluate the possible synergistic or antagonistic effect of these phenolic compounds between them and with other molecules present in the extracts.

#### 4.5.4. Prevention of Type 2 Diabetes and Obesity Beyond Inhibition of Digestive Enzymes

**Figure 2:**
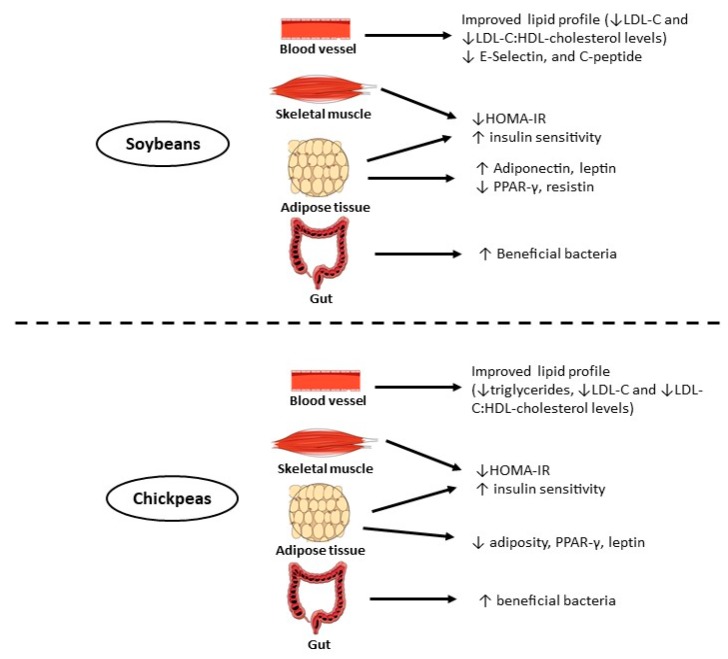
Proposed mechanisms of action for the reduction of insulin resistance by soybeans and chickpeas. Abbreviations: ↓, decrease; ↑, increase; PPAR-γ, peroxisome proliferator-activated receptor-γ; HOMA-IR, homeostasis model assessment-insulin resistance; LDL-c: low-density lipoprotein cholesterol; HDL: high-density lipoprotein.

The evidence about chickpea in relation to obesity and diabetes prevention have mainly been focused on the impact of the bioactive phenolic compounds (phenolic acids and flavonoids), bioactive peptides, carotenoids, phytoesterols, and fermentable fibers [47]. In addition, soybean, chickpea, and other legumes have a low glycemic index, which is important for patients with type 2 diabetes [263]. The proposed mechanisms of action of soybean, chickpea, and their bioactive compounds on insulin resistance, obesity, and lipid metabolism are summarized in Figure 2 and Table 9.

Leptin, a hormone produced by adipose cells, modulates energy balance and inhibits hunger. A decreased sensitivity to leptin in obesity jeopardizes detection of satiety even when high levels of this hormone and high energy stores occur. Among the mechanisms listed in Figure 2, human evidence on the role of soybean isoflavones in modulating sensitivity to leptin is still inconclusive [264,265,266] and needs further investigation. Furthermore, to the best of our knowledge, there is still a gap in the available literature with respect to the effects of isoflavones from chickpea.

Supplementation with soybean, chickpea, or their respective isoflavones can attenuate insulin resistance, possibly by reducing adiposity [94,185]. Furthermore, non-alcoholic fatty liver disease (NAFLD) is associated with an increase in the prevalence of obesity and diabetes [267]. The effects of soy isoflavones on the development of NAFLD in animal models, as well as the mechanisms involved (e.g., lipid metabolism, oxidative stress, and inflammation), were summarized by Van De Wier et al. [268]. According to these authors, histological studies related to inflammation support the anti-inflammatory effect of isoflavones. Finally, the same report suggests that clinical trials on the use of soy isoflavones in NAFLD patients are still necessary.

Yang et al. [185] showed that chickpea attenuated hyperglycemic, hyperinsulinemic, visceral adiposity, and lipid accumulation in rats induced by a chronic high-fat diet. Animal studies have shown that soy protein and soy isoflavones could improve insulin sensitivity and glucose control [269,270,271], and population studies suggested a lower risk of insulin resistance and type 2 diabetes [272,273]. A meta-analysis suggested an inverse association between soy food consumption (soy protein and isoflavones) and risk of type 2 diabetes mellitus [274]. Post-menopausal women who consumed a high soy diet had a lower body mass index and fasting insulin [275]. These protective effects can be found, especially, in women probably because phytoestrogen is structurally similar to female hormone estradiol with receptor-mediated estrogenic activity, and probably may affect insulin modulation [274].

Several in vivo studies have reported the beneficial effect of daidzein and genistein intake in the glycemic regulation among different animal models. A high-fat diet supplemented with soy isoflavones (0.4%, *w*/*w*) reduced weight gain, improved glucose metabolism and insulin sensitivity, reduced hepatic lipid accumulation, and increased gene expression of Cpt1α and Acox-1 associated with lipid oxidation in C57BL/6J mice [276]. Genistein and daidzein showed to exert anti-diabetic effects by modulating hepatic glucose (glucokinase, glucose-6-phosphate dehydrogenase) and lipid (fatty acid synthase, carnitine palmitoyltransferase, β-oxidation), regulating enzyme activities in db/db mice [277]. Genistein and daidzein treatment for 9 weeks caused a reduction in fasting blood glucose with concomitant decrease in plasma insulin and C peptide levels by down-regulating glucose-6-phosphate dehydrogenase (G6PD), phosphoenolpyruvate carboxykinase, fatty acid β-oxidation, and carnitine palmitoyltransferase activities, while up-regulating malic enzyme and G6PD activities in liver with preservation of pancreatic β-cells in non-obese diabetic mice [278].

Many studies have reported regulatory mechanisms of isoflavones on glucose tolerance, insulin secretion, and pancreatic β-cells in streptozotocin (STZ)-induced diabetic rats. El-Kordy and Alshahrani [285] demonstrated a protective and regenerative effect of genistein on pancreatic β-cell damage and improvement in serum levels of insulin and glucose in STZ-induced diabetic rats in a dose-dependent manner. Isolated soy protein and genistein were shown to regulate hyperglycemia in STZ-induced diabetic rats [152]. Another study also demonstrated the effect of genistein on glycemia, glucose tolerance, and insulin levels in STZ-induced diabetic mice [286]. Wright et al. [287] suggested that genistein has a direct inhibitory effect on GLUT4 in rat adipocytes and soleus muscles.

Dietary soy isoflavone supplementation (genistein, daidzein, and glycitein 0.1% *w*/*w*) reduced fat deposition in an animal model of obesity and diabetes [282]. Administration of soybean isoflavones (150 and 450 mg/kg) in rats lowered fasting insulin levels and HOMA-IR, reduced total white adipose tissue weight, lowered plasma resistin levels, and increased circulating protein and mRNA levels of adiponectin in perirenal white adipose tissue compared to the insulin resistant control group [283]. Furthermore, according to the same study [283], the higher supplementation dose of soy isoflavones (450 mg/kg/day) increased circulating protein and adipose mRNA levels of leptin and lowered adipose mRNA levels of resistin compared to the insulin resistant control group.

Higher risk for obesity and insulin resistance due to the decline of estrogen has been reported in postmenopausal women [288]. However, phytoestrogens (e.g., isoflavones) may reduce the risk of these metabolic diseases. Accordingly, Choi et al. [268] investigated the effect of genistein supplementation (0.1%, *w*/*w*) in a high-fat diet ovariectomized rats. Treated rats showed smaller-sized adipocytes and decreased HOMA-IR index, reaching levels comparable to those of the non-ovariectomized group [288]. In addition, genistein supplementation resulted in the reduction of hepatic fatty acid synthase activity, fatty acid synthesis genes, and showed an up-regulation in carnitine palmitoyltransferase, β-oxidation, succinate dehydrogenase activity, and fat utilization genes [288].

Peroxisome proliferator-activated receptor (PPAR) and their subtypes (alpha, beta, gamma) are related to insulin sensitivity [289]. PPAR-γ from adipose tissue indirectly modulates glucose and lipid homeostasis due to its regulation of adipocyte differentiation [290]. It has been suggested that chickpeas and soybeans isoflavones can down-regulate PPAR-γ, preventing the development of large and dysfunctional adipocytes that occurs in obesity and insulin resistance [291]. Luo et al. [276] evaluated the effect of soy isoflavones (genistein and daidzein) on C57BL/6J mice fed with a low-fat diet, western diet (WD), WD + 0.16% (*w*/*w*) of genistein or daidzein. Genistein and daidzein decreased food intake, body weight gain and induced liver X Receptor (LXR)-mediated pathways. Furthermore, a higher reduction of energy intake (26%) was found in genistein-treated mice compared to that of daidzein (8%). According to these authors, the reduction of food intake may stem from the activation of transcription factor PPARα due to genistein and daidzein intake. PPARα agonist transiently decreases the intake of food. In addition, reduced food intake associated with PPARα agonist treatment may be associated with cholecystokinin (CCK)-A receptor production. CCK is secreted from duodenal and jejunal mucosal cells in response to the intake of fat and protein. CCK has several physiological effects, including slowing gastric emptying and suppressing energy intake.

Phosphoenolpyruvate carboxykinase (PEPCK) activity regulate normal blood glucose levels and has been shown to be increased in diabetic animal models. Dkhar et al. [284] investigated the effect of genistein on the expression of PEPCK and glucose production in alloxan-induced diabetic mice. Genistein (50 mg/kg body weight) reduced fasting glucose levels, PEPCK-C expression and increased AMPK and ERK^1/2^ phosphorylation states in the liver of the genistein-treated alloxan-induced diabetic mice [284]. The same study also showed that glucose production in HepG2 cells was reduced by about 50% in cells treated with genistein (30 µM).

Epidemiological and prospective studies have shown the beneficial effects of soy-foods consumption in lowering the incidence of diabetes type 2 [292,293,294]. A randomized, cross-over, double-blinded trial of dietary supplementation with soy phytoestrogens in postmenopausal women resulted in lower values for fasting insulin, insulin resistance, and glycated hemoglobin A1c, when compared with placebo [190]. Soybean inclusion in the diet decreased insulin resistance and fasting plasma glucose in a randomized cross-over clinical trial with postmenopausal women with metabolic syndrome [191]. Li et al. [281] compared the effects of a soy-based meal replacement versus an individualized diet plan on weight loss and metabolic profile in diabetic patients randomized to treatment. The soy-based group underwent a greater weight loss, fasting plasma glucose (6 months), glycated hemoglobin A1c, and high sensitivity C-reactive protein. Furthermore, a greater number of subjects in a soy-based meal replacement group reduced their use of sulfonylureas and metformin as compared to the individualized diet plan. Medications, such as anti-hyperglycemic, are provided by the government in some places like Brazil [295]. Therefore, by improving health and reducing the use of anti-hyperglycemic medications, the inclusion of functional foods in the diet may also be helpful to overcome an eventual economic burden.

High serum concentrations of soluble E-selectin have been found to correlate with obesity in patients with type 2 diabetes mellitus [169]. Human intervention studies focusing on the health effects of chickpea and its components are also available in the literature. Incorporation of chickpeas in the diet reduced serum total fasting insulin concentration and HOMA-IR in healthy subjects [187]. Chickpea-based meal led to a lower glucose and insulin response in an acute consumption compared with a wheat-based meal [280]. A systematic review and meta-analysis of randomized controlled experimental trials in people with and without diabetes were carried out by Sievenpiper et al. [296]. According to these authors, pulses alone or in low-glycemic-index or high-fiber diets improve markers of longer-term glycemic control in humans.

Polyphenols can also play a role by modulating gut microbiota in such a prebiotic-like effect [23,297]. In addition, the literature has shown that the addition of excess fructooligosaccharide (FOS) may preserve genistein in human gut microflora in vitro [298]. Guadamuro [299] suggested that isoflavones may act as an alternative energy source, thus increasing the production of equol and short-chain fatty acids (SCFA), which are pivotal bacterial metabolites related to intestinal health benefits. The possible role of SCFA on specific receptors that influence gut epithelial, enteroendocrine, and pancreatic β-cells, as well as blood and renal vessels, adipose tissue, and peripheral nervous system, has been summarized by Li et al. [300]. Therefore, as sources of isoflavones, chickpea and soybean may promote the secretion of SCFA and, hence, their potential benefits do not rely only on the release of bioavailable isoflavones in the plasma and other organs but also in indirectly mediating receptors related to several chronic ailments.

## 5. Conclusions

Isoflavones as aglycones are usually more bioavailable than their conjugated counterparts. Furthermore, the literature has supported their higher antioxidant activity compared to that of their conjugates. The contents of daidzein and genistein in chickpeas are comparable to the concentrations reported in soybeans, which suggests that this legume seed may potentially substitute soybeans as a source of isoflavone aglycones. Contrary to soybean, there is very little information in the literature on the role of genetics with respect to the polyphenolic composition of chickpea, and, to the best of our knowledge, there is no study associated with the genetic regulation of isoflavones in chickpea seeds. Much has been discussed about the role of oxidative stress and inflammation in several chronic ailments, and we have demonstrated that both legumes show antioxidant and anti-inflammatory potential. Hence, their ability in preventing cardiovascular diseases and certain types of cancer appears to be irrefutable. In contrast, due to their lower lipid content, chickpea seems to offer a better option in weight management and prevention of type 2 diabetes. Several biomarkers related to these two diseases were discussed, and the effect of isoflavones in inhibiting digestive enzymes must be better clarified. Likewise, among the mechanisms discussed, human evidence on the role of soybean isoflavones in modulating sensitivity to leptin is still inconclusive, which warrants further investigation.

## Figures and Tables

**Table 1:** Proximate composition (g/100 g) of chickpea and soybean.

Component	Chickpea	Soybean	References
Ash	2.54–3.90	4.42–6.29	[48,49,50,51,52]
Lipid	1.12–6.80	14.9–23.3	[48,49,50,51,52,53,54]
Protein	18.3–25.2	36.3–47.0	[48,49,50,51,52,53,54]
Soluble fiber	1.23–1.38	9.05–9.33	[50,52]
Insoluble fiber	14.1–23.2	18.2–21.2	[50,52]

**Table 2:** Total phenolic contents (mg GAE/g) of chickpea and soybeans.

Feedstock	Organic Solvent *			References
	Acetone	Ethanol	Methanol	
Chickpea	1.44–1.81	0.93–1.54	8.02–10.84	[124,125,126,127,128]
Soybean	0.98–2.62	2.04–6.10	2.10–2.31	[121,129,130,131,132]

* Only the organic solvent is mentioned. The concentration in water varies among different studies.

**Table 3:** Isoflavone profile (%) of chickpea and soybean *.

Compound	Chickpea	Soybean	Method	Reference
Formononetin	2.61–16.6	nd	UPLC-ESI-Q-TOF-MS, HPLC/MS	[94,134]
Biochanin A	17.8–30.0	nd	UPLC-ESI-Q-TOF-MS, HPLC-ESI/MS	[94,135]
Biochanin glucoside	13.3–29.1	nd	UPLC-ESI-Q-TOF-MS	[94]
Daidzein	0.30–20.0	0.32–7.65	HPLC, UPLC-ESI-Q-TOF-MS, UPLC-ESI-Q-TOF-MS	[94,130,132,134]
Daidzin	nd	8.73–21.3	UPLC-ESI-Q-TOF-MS	[130,132]
Malonyldaidzin	nd	20.5–28.1	HPLC, UPLC-ESI-Q-TOF-MS, UPLC-ESI-Q-TOF-MS	[130,132]
Genistein	10.0–25.5	0.26–1.00	HPLC, UPLC-ESI-Q-TOF-MS, UPLC-ESI-Q-TOF-MS, HPLC-ESI/MS	[94,130,132,135]
Genistin	nd	7.73–23.8	HPLC, UPLC-ESI-Q-TOF-MS	[130,132]
Malonylgenistin	nd	36.0–42.5	HPLC, UPLC-ESI-Q-TOF-MS	[130,132,136]
Glycitein	nd	0.78–0.97	UPLC-ESI-Q-TOF-MS	[132,135]
Glycitin	nd	3.14–4.51	HPLC, UPLC-ESI-Q-TOF-MS	[130,132]
Malonylglycitin	nd	1.93–7.98	HPLC; UPLC-ESI-Q-TOF-MS	[130,132]

Abbreviations: HPLC, high-performance liquid chromatography; UPLC, ultra-performance liquid chromatography; ESI, electrospray ionization; Q, quadrupole; TOF, time of flight. * MS (mass spectrometry) may contemplate tandem mass spectrometry (MS^n^).

**Table 4:** Scavenging of peroxyl radical and reducing power of chickpea and soybean.

Method	Chickpea	Soybean	Reference
ORAC (µmol TE/g)	8.74–52.2	22.2–86.8	[93,121,125,128,144]
FRAP (mmol Fe^2+^/g)	0.73–1.13	1.24–1.96	[121,128,145]

Abbreviations: ORAC, oxygen radical absorbance capacity; FRAP, Ferric reducing antioxidant power; TE, Trolox equivalents.

**Table 5:** Inhibition of α-glucosidase by soybean and chickpea phenolic extracts.

Extraction	Identification	Methods	Main Findings	Reference
Chickpea 80% ethanol for free phenolics, NaOH hydrolysis for bound phenolics	Free (93 mg GAE/100 g sample) and bound (128 mg GAE/100 g sample) phenolic compounds	α-glucosidase inhibition index (GI = control activity/sample activity)	GI of 1.23 and 1.71 for free and bound phenolic extracts, respectively. Incubation of chickpea flour with *Rhizopus oligosporus* increased GI.	[125]
Chickpea 80% methanol with 1% HCl	Phenolic acids: ferulic, *p*-hydroxybenzoic, protocatechuic, caffeic, chlorogenic, and *p*-coumaric. Flavonols: quercetin, kaempferol, myricetin. Isoflavones: daidzein, genistein, and genistein hexoside Proanthocyanidins	Enzyme inhibition (IC_50_)	IC_50_ = 92.2 µg/mL. Lower activity compared to underutilized Indian legumes (horse gram and cowpea).	[119,127]
Chickpea 70% ethanol	Ferulic acid (9.1 mg/100 g), *p*-coumaric (4.5 mg/100 g), sinapic acid (4.5 mg/100 g), chlorogenic acid (0.13 mg/100 g)	Enzyme inhibition (%), enzyme source not specified	15.9% inhibition. Lower inhibition compared to beans and peas, but higher than soybean. No correlation with phenolic acids nor antioxidant capacity.	[131]
Chickpea 80% ethanol for free phenolics, NaOH hydrolysis for bound phenolics	Free (72 mg GAE/100 g sample) and bound (105 mg GAE/100 g sample) phenolic compounds	Enzyme inhibition (%), enzyme source not specified	26 and 76% inhibition for free and bound phenolic extracts, respectively, obtained from brownies baked with chickpea flour.	[241]
Chickpea 85% methanol	Total phenolic compounds, total flavonoids, total anthocyanins.	Rat intestinal α-glucosidase inhibition (%)	Between 16.2 and 43.6% inhibition. No correlation with and phenolic content nor antioxidant capacity. Germination increased inhibition.	[238]
Soybean free (80% acetone) and bound (4 M NaOH) phenol extracts	Free (98 mg GAE/100 g sample) and bound (77 mg GAE/100 g sample)	Enzyme inhibition (IC_50_)	IC_50_ = 373 and 458 µg/mL for free and bound phenolic compounds, respectively.	[129]
Solid state fermentation (SSF) and germinated soybean water extracts	3–5 mg catechin equivalents (CE)/g for dry soybeans, 6–7 mg CE/g SSF, 4.5 mg CE/g sprouted soybean	α-glucosidase (yeast) inhibition index (GI = control activity/sample activity)	Approximately 1.1 GI for SSF and sprouted extracts. Less inhibition compared to α-amylase.	[237]
Soybean 70% ethanol	Ferulic acid (14.69 mg/100 g), sinapic acid (5.41 mg/100 g), *p*-coumaric (1.45 mg/100 g), caffeic acid 0.63 mg/100 g	Enzyme inhibition (%), enzyme source not specified	12.06% inhibition. Lower inhibition compared to chickpea, beans, and peas. No correlation with phenolic acids nor antioxidant capacity.	[131]
Acetone: water: acetic acid soybean extract. Fractionation of the extract with Amberlite XAD-7 resin	Total phenolic compounds, total flavonoids, total condensed tannins	Enzyme inhibition (IC_50_)	IC_50_ = 75.4 and 5.4 µg/mL for crude and tannin-rich extracts, respectively. Lower inhibition than black bean extracts.	[242]
70% methanol soybean phenolic extract	Total phenolic compounds (2.1 mg GAE/g). Glucosylated, molonylated, acetylated, and aglycone isoflavones: daidzein, genistein, and glycitein	Enzyme inhibition (%) of 1 mg/mL extract solution	Different methanol-water extracts were obtained. 70% of methanol showed the highest phenolic, isoflavone content, and enzyme inhibition (81%).	[132]
70% methanol hydroxylated and fermented soybean milk phenolic extract	Total phenols (3.7–6.6 mg GAE/g DW), total flavonoids (0.1–0.3 mg RE/g DW). Glucosylated and aglycone isoflavones: daidzein, genistein, and glycitein	Enzyme inhibition (%) of 0.5 mg/mL extract solution	10 different varieties of soybean were analyzed. Enzyme inhibition between 32.4–55.5%. Glucosylated isoflavones decreased, and aglycones increased.	[239]
Fermented soybean water extract	Total phenolic compounds (22.2–30.7 mg GAE/g)	Enzyme inhibition (IC_50_)	Different fungal strains. IC50 ranged between 27.4 and 41.0 mg/mL.	[240]
Solid state fermentation (SSF) and germinated soybean water extracts	3–5 mg catechin equivalents/g DW for dry soybeans, 6–7 mg CE/g DW SSF, 4.5 mg CE/g DW sprouted soybean	α-glucosidase (yeast) inhibition index (GI = control activity/sample activity)	Approximately 1.5–2.08 AI for SSF extracts and 1.3 AI for sprouted samples.	[237]

Abbreviations: GAE, gallic acid equivalent; CE, catechin equivalent; RE, rutin equivalent; DW, dry weight. IC_50_, the concentration necessary to inhibit enzymatic activity by 50%.

**Table 6:** Inhibition of α-amylase by soybean and chickpea phenolic extracts.

Extraction	Identification	Methods	Main Findings	Reference
Chickpea 80% ethanol for free phenolics, NaOH hydrolysis for bound phenolics	Free (93 mg GAE/100 g sample) and bound (128 mg GAE/100 g sample) phenolic compounds	α-amylase inhibition index (AI = control activity/sample activity)	AI of 1.9 and 1.66 for free and bound phenolic extracts, respectively. Incubation of chickpea flour with *Rhizopus oligosporus* increased AI. Good correlation between phenolics and AI.	[125]
Chickpea 80% methanol with 1% HCl	Phenolic acids: ferulic, *p*-hydroxybenzoic, protocatechuic, caffeic, chlorogenic, and *p*-coumaric. Flavonols: quercetin, kaempferol, myricetin. Isoflavones: daidzein, genistein, and genistein hexoside Proanthocyanidins	Enzyme inhibition (IC_50_)	IC_50_ = 108.3 µg/mL.	[119,127]
Chickpea water extract	Not identified (peptides and/or phenolic compounds)	Enzyme inhibition (%)	34% inhibition. Lower inhibition compared to fava beans, peas, and lentils and similar to beans. Activity increased by processing.	[245]
Chickpea water extract	Not identified (probably peptides)	Enzyme inhibition (%)	No inhibition. High activity in beans and soybean, reduced by soaking and cooking.	[243]
Chickpea protein extracts	Not identified (probably peptides)	Enzyme inhibition (Units/g)	16 different varieties evaluated for antinutritional factors. 0–15 Units/g, average 8.7. Activity lower than other pulses decreases by cooking.	[244]
Soybean free (80% acetone) and bound (4 M NaOH) phenol extracts	Free (98 mg GAE/100 g sample) and bound (77 mg GAE/100 g sample)	Enzyme inhibition (IC_50_)	IC_50_ = 526 and 320 µg/mL for free and bound phenolic compounds, respectively.	[129]
Acetone: water: acetic acid soybean extract. Fractionation of the extract with Amberlite XAD-7 resin	Total phenolic compounds, total flavonoids, total condensed tannins	Enzyme inhibition (IC_50_)	IC_50_ = 2.25 and 0.25 mg/mL for crude and tannin-rich extracts, respectively. Higher inhibition than black bean extracts.	[242]
Water soybean phenolic extract	Not identified (probably peptides)	Enzyme inhibition (Units/g)	939 and 899 units/g dry weight for raw and soaked soybeans, respectively. No inhibitory activity for cooked samples.	[243]
Fermented soybean water extracts	Total phenolic compounds (2–12.7 mg CE/g DW), glucosidated and aglycone isoflavones: daidzein, genistein	Enzyme activity (units/g DW)	α-amylase activity increased during fermentation (39.3–128.2 units/g DW) depending on fermentation time and Bacillus species.	[246]

Abbreviations: GAE, gallic acid equivalent; CE, catechin equivalent; RE, rutin equivalent; DW, dry weight. IC_50_, the concentration necessary to inhibit enzymatic activity by 50%.

**Table 7:** Inhibition of α-glucosidase by soybean and chickpea phenolic extracts.

Extraction	Identification	Methods	Main Findings	Reference
Chickpea 80% methanol with 1% HCl	Not identified	Enzyme inhibition (IC_50_)	IC_50_ extract: 6.3 mg/mL. Higher activity than other pulses. No effect of in vitro digestion.	[247]
Fermented soybean supplements 80% methanol extract	75 ppm GAE non-fermented extract. 160 ppm GAE 60 h fermented extract. Aglycone flavonoids and isoflavones	Enzyme activity (% of control). 10 mg/mL extracts	Fermentation increased lipase inhibition and content of aglycone flavonoids and isoflavones.	[248]
Soybean 80% methanol with 1% HCl	Not identified	Enzyme inhibition (IC_50_)	IC_50_ extract: 6.65–6.97 mg/mL. Similar activity to chickpea.	[247]
Acetone: water: acetic acid soybean extract. Fractionation of the extract with Amberlite XAD-7 resin	Total phenolic compounds, total flavonoids, total condensed tannins	Enzyme inhibition (IC_50_)	IC_50_ = 0.27 and 0.081 mg/mL for crude and flavonoids rich extracts, respectively.	[242]
70% methanol hydroxylated and fermented soybean milk phenolic extract	Total phenols (3.7–6.6 mg GAE/g DW), total flavonoids (0.1–0.3 mg RE/g DW). Glucosylated and aglycone isoflavones: daidzein, genistein, and glycitein	Enzyme inhibition (%) of 0.5 mg/mL extract solution	10 different varieties of soybean were analyzed. Enzyme inhibition between ND–43.4%. Glucosylated isoflavones decreased, and aglycones increased.	[239]

Abbreviations: GAE, gallic acid equivalent; RE, rutin equivalent; DW, dry weight. IC_50_, the concentration necessary to inhibit enzymatic activity by 50%.

**Table 8:** Structure-activity relationship of flavonoids-digestive enzymes inhibition.

Phenolics	Structure	α-amylase		α-glucosidase		Lipase
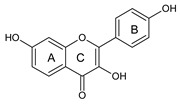	Ring A	Flav	Iso	Flav	Iso	Flav
	-OH	↑	↑	↑	↑	↑
	-Gly	↓		↓		↓
	-OMe	↓		↓	↓	
	Ring C					
	C2=C3	↑		↑		↑
**Flavonoid**	-OH	↑		↑		↑
	C4=O	↑		↑		↑
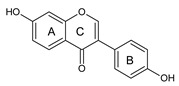	-OMe			↓	↓	
	-OGalloyl	↑		↑		↑
	-Gly	↓		↓	↓	↕
	Ring B					
	O-Me	↓		↑	↑	
**Isoflavone**	-OH	↑		↑	↕	
	-Gly	↓		↓		

Flav: Flavonoid. Iso: Isoflavone. -Gly: Glycosylation. -OGalloyl: attachment of a galloyl moiety to the hydroxyl group. ↑ increase in enzymatic inhibition. ↓ decrease in enzymatic inhibition. ↕ No systematic effect; it can increase or decrease the inhibitory activity depending on the flavonoid.

**Table 9:** Chickpea, soybean, and isoflavones in the prevention of type 2 diabetes and obesity and associated metabolic biomarkers in vivo.

Model	Treatment (Dose; Duration)	Main Findings	Reference
**Chickpea**			
Animal	Male rats were fed a standard; HFD; or an HFD plus 10% raw crushed chickpea seeds diet for 8 months	Chickpeas: ↓ HOMA-IR, postprandial hyperglycemia, and hyperinsulinemia ↓ body and epididymal adipose tissues weight - Improvement in the lipid profile (↓triacylglycerols, ↓LDL-c, and ↓LDL-c:HDL-c levels) ↓ leptin mRNA levels in epididymal adipose	[185]
Clinical trial	Hypocaloric balanced diet + 4 servings/week of non-soybean legumes (lentils, chickpeas, peas, and faba beans) for 8 weeks in obese subjects	- Weight loss ↓ total cholesterol ↓ MDA	[279]
Clinical trial	Inclusion of 728 g chickpea per week for 12 weeks in healthy subjects in a crossover design	↓ serum total cholesterol	[187]
Clinical trial	Chickpea diet (140 g of canned, drained chickpeas, chickpea bread, and chickpea biscuits) for 5 weeks in hypercholesterolemic subjects in a randomized crossover design	↓ serum LDL-c and total cholesterol concentrations	[186]
Clinical trial	Randomized crossover design in healthy subjects: - Acute: 200 g chickpea (cooked and drained)	↓ glucose and insulin responses acutely	[280]
Clinical trial	Inclusion of 728 g chickpea per week for 12 weeks in healthy subjects in a crossover design	↓ fasting insulin concentration and HOMA-IR	[187]
**Soybean**			
Clinical trial	70 patients with type 2 diabetes were randomly divided: test group (35 people) with 60 g soy nut and control group (35 people) under the usual diet of diabetes for 8 weeks	↓ fasting blood glucose ↓ total serum cholesterol, LDL-c, and E-Selectin ↑ the capacity of serum total antioxidants	[189]
Clinical trial	Randomized, double-blind, cross-over trial with 32 postmenopausal women with diet-controlled type 2 diabetes, supplemented with soy (30 g protein/day, 132 mg isoflavones/day) for 12 weeks	↓ fasting insulin, insulin resistance, glycated hemoglobin ↓ total cholesterol, LDL-c, cholesterol/HDL-c ratio - No differences in HDL-c, triacylglycerols, weight, blood pressure	[190]
Clinical trial	Randomized crossover clinical trial with 42 postmenopausal women with metabolic syndrome. Participants assigned to consume a control diet, a soy-protein diet, or a soy-nut diet each for 8 weeks.	↓ insulin resistance, fasting plasma glucose ↓ LDL-c ↓ serum C-peptide	[191]
Clinical trial	Diabetic patients (n = 77) were randomized prospective to the two treatments for 12 months: soy-based meal replacement, or individualized diet plan	Soy-based meal replacement presented greater values compared to the individualized diet plan: - ↓ weight - ↓ fasting plasma glucose at 6 months - ↓ glycated hemoglobin A1c - ↓ medications - ↓ high sensitivity C-reactive protein at 6 months	[281]
Animal	Soy isoflavone supplementation (0.1% *w*/*w*) of lean and obese spontaneously hypertensive rat/N-corpulent rats	Soy isoflavone: ↓ body weight of obese rats ↓ peri-renal, epididymal, and subdiaphragmatic fat pad weights in lean and obese rats ↓ ileal fat pads in obese rats	[282]
Animal	HFD-induced insulin resistant rats treated with soy isoflavone with three different dosages (50 mg, 150 mg, and 450 mg/kg/day) for 30 days	↓ fat pad weights ↓ fasting insulin and HOMA-IR ↑ plasma and mRNA adiponectin and leptin levels, ↓ resistin levels	[283]
**Genistein and daidzein**			
Animal	Healthy normal mice divided into groups and intraperitoneally administered: dimethyl sulfoxide (DMSO) (control group) and genistein (50 mg/kg + 10% DMSO). Alloxan-induced diabetic male mice were treated with DMSO 10% and genistein (50 mg/kg + 10% DMSO)	Genistein: ↓ fasting glucose levels ↓ PEPCK-C expression ↑ AMPK and ERK ½ phosphorylation states in the liver	[284]
Animal	C57BL/6J mice were fed: low-fat diet; western-style diet, and western-style diet + 0.16% (*w*/*w*) of genistein or daidzein for 10 weeks.	Genistein and daidzein: ↓ food intake ↓ body weight gain - Induced LXR-mediated pathways	[276]

Abbreviations: ↓, decrease; ↑, increase; HFD, high-fat diet; HOMA-IR, homeostasis model assessment-insulin resistance; MDA, malondialdehyde; PEPCK-C, Phosphoenolpyruvate carboxykinase; AMPK, activated protein kinase; ERK ½, extracellular signal-regulated kinase ½; LXR, Liver X Receptor.
